# Atorvastatin inhibits the immediate-early response gene EGR1 and improves the functional pro of CD4^+^T-lymphocytes in acute coronary syndromes

**DOI:** 10.18632/oncotarget.15420

**Published:** 2017-02-16

**Authors:** Anna Severino, Chiara Zara, Mara Campioni, Davide Flego, Giulia Angelini, Daniela Pedicino, Ada Francesca Giglio, Francesco Trotta, Simona Giubilato, Vincenzo Pazzano, Claudia Lucci, Antonio Iaconelli, Aureliano Ruggio, Luigi Marzio Biasucci, Filippo Crea, Giovanna Liuzzo

**Affiliations:** ^1^ Institute of Cardiology, Catholic University, Rome, Italy

**Keywords:** acute coronary syndromes, T-lymphocytes, transcription factors, statins, inflammation, Pathology Section

## Abstract

Background- Adaptive immune-response is associated with a worse outcome in acute coronary syndromes. Statins have anti-inflammatory activity beyond lowering lipid levels. We investigated the effects of *ex-vivo* and *in-vivo* atorvastatin treatment in acute coronary syndromes on CD4^+^T-cells, and the underlying molecular mechanisms.

Approach and results- Blood samples were collected from 50 statin-naïve acute coronary syndrome patients. We assessed CD4^+^T-cell activation by flow-cytometry, the expression of 84 T-helper transcription-factors and 84 T-cell related genes by RT-qPCR, and protein expression by Western-blot, before and after 24-hours incubation with increasing doses of atorvastatin: 3-10-26 g/ml (corresponding to blood levels achieved with doses of 10-40-80 mg, respectively). After incubation, we found a significant decrease in interferon-?-producing CD4^+^CD28^null^T-cells (*P* = 0.009) and a significant increase in interleukin-10-producing CD4^+^CD25^high^T-cells (*P* < 0.001). Atorvastatin increased the expression of 2 genes and decreased the expression of 12 genes (in particular, EGR1, FOS,CCR2 and toll like receptor-4; >3-fold changes).

The *in-vivo* effects of atorvastatin were analyzed in 10 statin-free acute coronary syndrome patients at baseline, and after 24h and 48h of atorvastatin therapy (80 mg/daily): EGR1-gene expression decreased at 24h (*P* = 0.01) and 48h (*P* = 0.005); EGR1-protein levels decreased at 48h (*P* = 0.03).

Conclusions-In acute coronary syndromes, the effects of atorvastatin on immune system might be partially related to the inhibition of the master regulator gene EGR1. Our finding might offer a causal explanation on why statins improve the early outcome in acute coronary syndromes.

## INTRODUCTION

Over the past few years our understanding of the importance of inflammation in coronary instability is considerably increased [1].

CD4^+^CD28^null^ T-cells are a subset of long-lived directly cytotoxic CD4^+^ T-lymphocytes producing large amount of the pro-inflammatory cytokine interferon-γ (IFN-γ), that have been implicated in the pathogenesis of various chronic inflammatory diseases. We previously demonstrated that circulating CD4^+^CD28^null^T-cell frequency higher than 4% increase the risk of acute coronary syndromes (ACS), particularly in diabetic patients [2, 3].

At the other extreme, naturally occurring regulatory T-cells are a major cellular source of interleukin (IL)-10, a potent anti-inflammatory cytokine. These regulatory T-cells are involved in the control of autoimmunity [4].Accordingly, a lower number or a decreased function of these cells has been found in patients suffering from lupus erythematous, type-1 diabetes, rheumatoid arthritis, and multiple sclerosis [5], as well as in patients with ACS [6, 7]. Moreover, in ACS the production of pro-inflammatory cytokines is not adequately counterbalanced by anti-inflammatory cytokines, such as IL-10; these alterations have been related to a worse short- and long-term prognosis [8, 9].We recently observed that a subset of ACS patients presents an alteration of the immune response, associated to a worse outcome and characterized by reduced regulatory T-cell response to effector T-cell expansion [10].

Statins have anti-inflammatory and immune-suppressive activity besides lowering lipids that may, at least partially, account for their outcome improvement in the setting of both acute and chronic ischemic heart disease [11, 12, 13]. In particular, statins attenuate T-cell activation and proliferation, inhibit pro-inflammatory cytokine secretion and enhance anti-inflammatory cytokine secretion [14, 15, 16]. Two observational retrospective studies of our group have shown that in ACS the use of statins was associated with reduced levels of CD4^+^CD28^null^T-cells [17, 2]. In a small number of ACS patients, rosuvastatin treatment for 6 weeks induced CD4^+^CD28^null^T-cell apoptosis [18]. Recent studies have also suggested that statins may enhance regulatory T-cell responses [19, 20, 21].

In the present study, we sought to investigate the effects of increasing doses of atorvastatin on phenotype and function of different CD4^+^T-cell subsets, obtained from 50 statin-naïve patients presenting with non-ST elevation (NSTE)-ACS and raised levels of CD4^+^CD28^null^T-cells. To explore the mechanisms by which atorvastatin might suppress the immune response in ACS, we analyzed by quantitative PCR array the expression of 84 transcription factors involved in the immune response and 84 genes related to the functional properties of different T-helper cell subsets.

Finally, we assessed the *in-vivo* effects of high-dose of atorvastatin (80 mg/daily) in ACS patients.

## RESULTS

**Figure 1:**
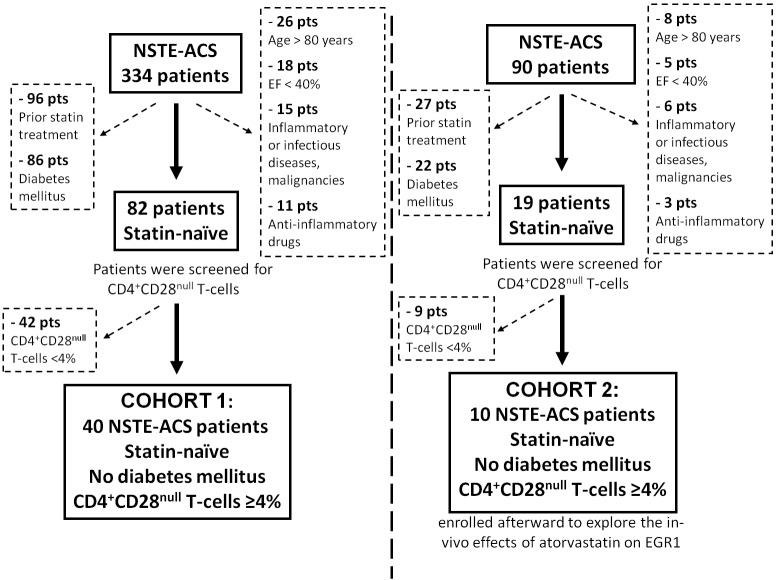
Flow diagram of patient selection and study design NST-ACS = Non ST elevation acute coronary syndrome; EF = left ventricular ejection fraction.

Patient selection and study design are presented in Figure-1.

Table 1 summarizes the clinical characteristics of the study population.

**Table 1:** Baseline characteristics of study population: 50 statin-naïve ACS patients

Age, mean ± SD (years)	64±12
Sex, n (F/M)	10/40
Clinical Presentation (UAIIIB/NSTEMI)	8/42
Smokers, n (%)	29 (58%)
Family History of CAD, n (%)	19 (38%)
Hypertension, n (%)	33 (66%)
Obesity, n (%)	10 (20%)
Dyslipidemia, n (%)	26 (52%)
Previous Cardiovascular Events, n (%)	7 (14%)
Previous PCI/CABG, n (%)	10/5 (20%/10%)
Multivessel disease, n (%)	23 (46%)
In-hospital PCI/CABG, n (%)	32/14 (64%/28%)
LVEF, mean ± SD (%)	51±0.12
Total-C, mean ± SD (mg/dl)	185.3±49.1
LDL-C, mean ± SD (mg/dl)	130.9±34.3
HDL-C, mean ± SD (mg/dl)	40.9±12.8
TG, mean ± SD (mg/dl)	142.8±85.1
Plasma glucose, mean ± SD (mg/dl)	114.2±39.1
Lymphocytes, median-range (103/ml)	1.65 (0.63-4.33)

ACS=acute coronary syndromes; UA=unstable angina; NSTEMI=non-ST elevation acute myocardial infarction; CAD=coronary artery disease; PCI=percutaneous coronary intervention; CABG=coronary artery by-pass graft; LVEF = left ventricular ejection fraction; Total-C = Total-Cholesterol; LDL-C = LDL-Cholesterol; HDL-C = HDL-Cholesterol; TG = triglycerides.

**Figure 2:**
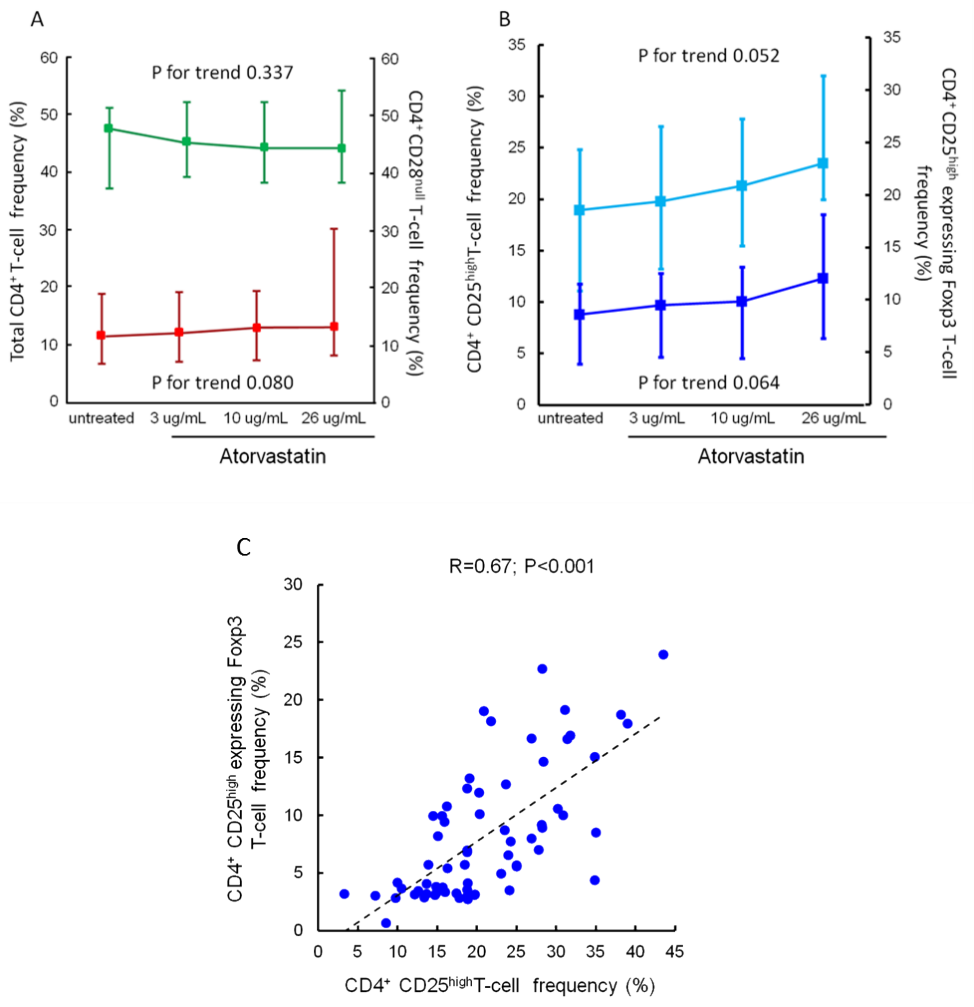
Effects of atorvastatin on total CD4 **^+^**T-cells, CD4**^+^**CD28**^null^**T-cells, CD4**^+^**CD25**^high^**T-cells and CD4**^+^**CD25**^high^** Foxp3**^+^**T-cells. Panel **A**. Frequencies of total CD4^+^ and of CD4^+^CD28^null^ T-cells were determined by flow-cytometry. CD4^+^T-cells were isolated from peripheral blood samples of 20 statin-naïve NST-ACS patients and incubated for 24 hours without and with increasing doses of atorvastatin. Data are presented as median and 95% CI. The percentage of both total CD4^+^ (indicated in green) and of CD4^+^CD28^null^ T-cells (indicated in red) did not change significantly after treatment with atorvastatin (P for trend = 0.337 and 0.080, respectively). Panel **B**. Frequencies of CD4^+^CD25^high^T-cells and of CD4^+^CD25^high^T-cells expressing the transcription factor Foxp3 were determined as described in Panel A. Data are presented as median and 95% CI. The percentage of both total CD4^+^CD25^high^T-cells (indicated in light blue) and of CD4^+^CD25^high^ Foxp3^+^ T-cells (indicated in dark blue) showed slight, but not statistically significant, changes after treatment with atorvastatin (P for trend = 0.052 and 0.064, respectively). Panel **C**. Correlation between CD4^+^CD25^high^T-cells and CD4^+^CD25^high^ Foxp3^+^T-cells. Frequencies of CD4^+^CD25^high^T-cells and of CD4^+^CD25^high^T-cells expressing the transcription factor Foxp3 were calculated as percentage of CD4^+^CD25^+^T-cell population. A significant correlation was observed among these T-cell subsets (R = 0.67; *P* < 0.001). Spearman rank correlation was performed on pooled data (untreated/treated with increased doses of atorvastatin).

The percentage of total CD4^+^T-cells, CD4^+^CD28^null^T-cells, CD4^+^CD25^high^T-cells and CD4^+^CD25^high^T-cells expressing the transcription factor Foxp3 did not change significantly after *ex-vivo* treatment with increasing doses of atorvastatin for 24 hours (Figure 2).

### Effects of atorvastatin on CD4^+^CD28^null^ T-cells and CD4^+^CD25^high^T-cells

**Figure 3:**
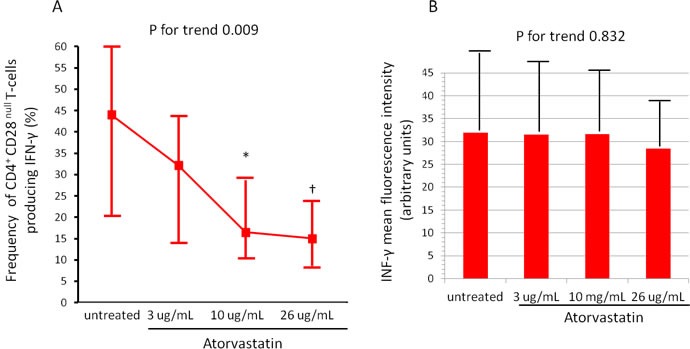
Effects of atorvastatin on CD4 **^+^**CD28**^null^** T-cells. CD4^+^T-cells were isolated from whole blood samples of 20 statin-naïve NST-ACS patients and incubated for 24 hours without and with increasing doses of atorvastatin. Cells were analyzed by flow-cytometry. **A**. The percentage of CD4^+^CD28^null^Tcells producing IFN-γ decreased after treatment with atorvastatin (P for trend = 0.009). Data are presented as median and 95% CI. *P = 0.014 untreated cells vs 10μg/mL of atorvastatin; †*P* = 0.006 untreated cells vs 26 μg/mL of atorvastatin. **B**. The mean fluorescence intensity (MFI) of intracellular IFN-γ expression by CD4^+^CD28^null^T-cells remained unchanged after atorvastatin treatment (P for trend = 0.832). Data are presented as mean±SD.

**Figure 4:**
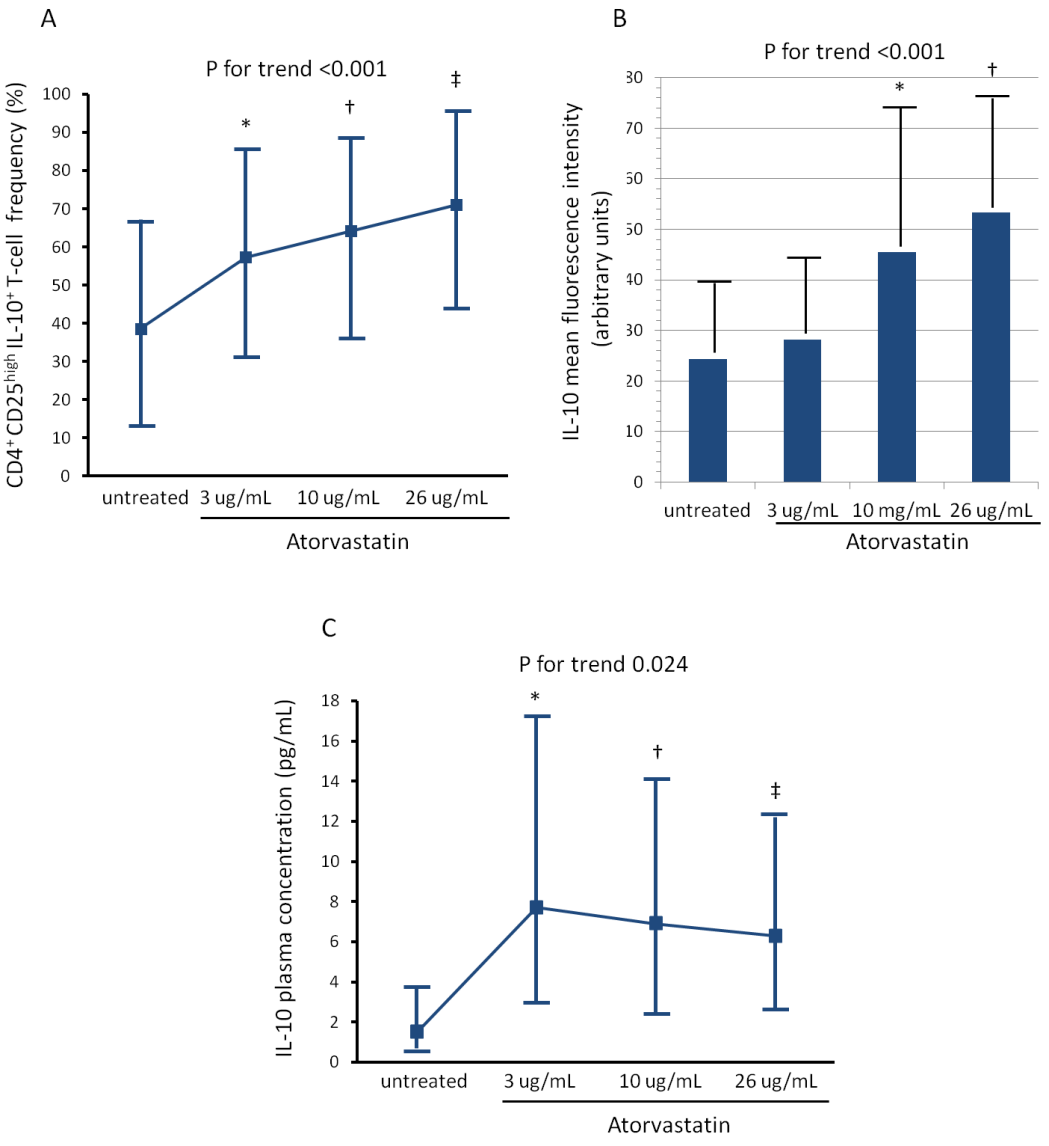
Effects of atorvastatin on CD4 **^+^**CD25**^high^**T-cells. Experimental conditions are reported in Figure 3. **A**. The percentage of CD4^+^CD25^high^Tcells producing IL-10 increased significantly after treatment with atorvastatin (P for trend < 0.001). Data are presented as median and 95% CI. *P = 0.034 untreated cells vs 3μg/mL of atorvastatin; †*P* = 0.022 untreated cells vs 10μg/mL of atorvastatin; ‡*P* < 0.001 untreated cells vs 26μg/mL of atorvastatin. **B**. The mean fluorescence intensity (MFI) of intracellular IL-10 expression by CD4^+^CD25^high^T-cells also increased significantly after atorvastatin treatment (P for trend < 0.001). Data are presented as mean±SD. *P = 0.056 untreated cells vs 10μg/mL of atorvastatin; †*P* < 0.001 untreated cells vs 26μg/mL of atorvastatin. **C**. IL-10 was measured by high-sensitivity ELISA in aliquots of 1mL of whole blood incubated for 24 hours without and with increasing doses of atorvastatin: 3-10-26μg/ml. IL-10 concentrations significantly increased after atorvastatin treatment (P for trend = 0.024). *P = 0.025 untreated cells vs 3μg/mL of atorvastatin; †*P* = 0.016 untreated cells vs 10μg/mL of atorvastatin; ‡*P* = 0.058 untreated cells vs 26ug/mL of atorvastatin.

The activation of CD4^+^CD28^null^T-cells and CD4^+^CD25^high^T-cell subset was modified by atorvastatin treatment. Indeed, the percentage of CD4^+^CD28^null^T-cells producing IFN-γ decreased from a median of 44.1% (range 20.5-60.9) (untreated cells) to 15.0% (range 8.6-23.8) after incubation with 26 μg/ml of atorvastatin (P for trend = 0.009) (Figure-3). Conversely, the percentage of CD4^+^CD25^high^T-cells producing IL-10 increased from a median of 38.6% (range 13.5-67.1) (untreated cells) to 71.1% (range 44.3-95.5), after incubation with 26 μg/ml of atorvastatin (P for trend < 0.001). Accordingly, the MFI of intracellular IL-10 expression increased after treatment (from 24.4±13.5 to 53.3±22.3; P for trend < 0.001) (Figure-4, panel A-B).

### Effects of atorvastatin on pro-inflammatory and anti-inflammatory cytokine concentrations

IL-10 concentration increased from a median of 1.5 pg/mL, range 0.7-39.9 (untreated blood) to 6.3 pg/mL (range 1-42.7) after incubation of whole blood samples, collected from an antecubital vein at the time of patient enrollment, with 26 μg/ml of atorvastatin (P for trend = 0.024) (Figure-4, Panel C). Although we used a high-sensitivity ELISA kit, IFN-γ was detectable in few patients at baseline and resulted undetectable after atorvastatin treatment (data not shown).

### Atorvastatin decreases the expression of key cellular pathways in ACS CD4^+^T-cells

To identify mechanisms by which atorvastatin might have immune-suppressive effects in CD4+T-cell populations, we analyzed the gene expression of a focused panel of 84 transcription factors downstream of signaling from cytokines, chemokines, growth factors, androgens and Toll-Like receptors. Then, we performed a PCR array profiling the expression of 84 genes including cytokine genes representative of the three classes of helper T-cells, genes encoding transcriptional factors that regulate the expression of these cytokines, markers of CD4+T-cell activation and other genes involved in the adaptive immune responses. PCR array analysis was applied on pooled RNA samples.

The complete list of genes investigated by PCR arrays, and their different expression after atorvastatin treatment, is reported in Table-2 and 3.

**Figure 5:**
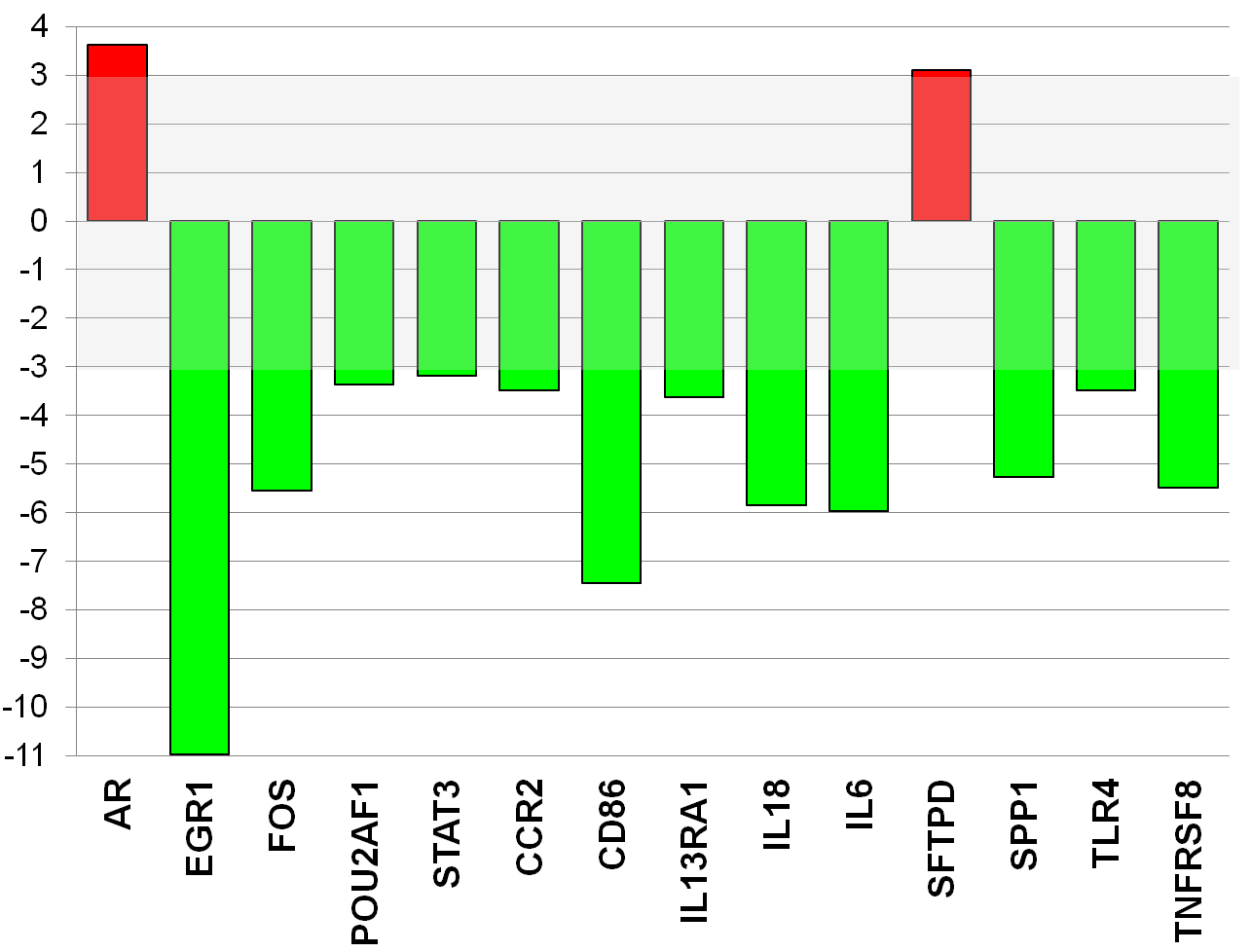
Atorvastatin decreases the expression of inflammatory genes and key transcription factors in CD4 **^+^**T-cells of patients with ACS. The expression of 84 transcription factors and 84 genes related to both T-effectors and T-regulatory cells was analyzed by quantitative PCR array using a pool of RNA (*n* = 20 patients). Data are presented as fold of regulation by atorvastatin treatment as compared with the gene expression in untreated CD4^+^T-cells. The expression of 2 genes was increased (red) and the expression of 12 genes was reduced (green) (>3 fold changes) by atorvastatin treatment (26 μg/ml for 24 hours) compared with control. The complete list of genes investigated by PCR arrays, and changes in their expression induced by atorvastatin, is reported in Tables 2 and 3.

PCR array analysis revealed that the expression of 2 genes was increased while the expression of 12 genes was decreased (>3-fold changes) by *ex-vivo* treatment of freshly isolated CD4^+^T-cells from ACS patients with a dose of 26 μg/ml atorvastatin for 24 hours compared with control (Figure-5). Among the transcription factors, atorvastatin decreased the expression of early growth response 1 (EGR1) and V-fos FBJ murine osteosarcoma viral oncogene homolog (FOS). Among the genes related to helper T-cell pathway, atorvastatin decreased the expression of chemokine (C-C motif) receptor-2 (CCR2), the Toll-like receptor-4 (TLR4), and the proinflammatory cytokines IL-6 and IL-18.

**Table 2:** Genes investigated by Human Transcription Factors RT^2^ Profiler™ PCR Array, and changes in their expression induced by atorvastatin

Position	Symbol	Description	Fold Regulation
**A01**	**AR**	**Androgen receptor**	**3,6217**
A02	ARNT	Aryl hydrocarbon receptor nuclear translocator	2,5198
A03	ATF1	Activating transcription factor 1	−1,3044
A04	ATF2	Activating transcription factor 2	−1,1277
A05	ATF3	Activating transcription factor 3	1,0473
A06	ATF4	Activating transcription factor 4 (tax-responsive enhancer element B67)	1,0329
A07	CEBPA	CCAAT/enhancer binding protein (C/EBP), alpha	−2,7959
A08	CEBPB	CCAAT/enhancer binding protein (C/EBP), beta	−1,4077
A09	CEBPG	CCAAT/enhancer binding protein (C/EBP), gamma	−1,4880
A10	CREB1	CAMP responsive element binding protein 1	−1,1277
A11	CREBBP	CREB binding protein	−1,3226
A12	CTNNB1	Catenin (cadherin-associated protein), beta 1, 88kDa	−1,6058
B01	DR1	Down-regulator of transcription 1, TBP-binding (negative cofactor 2)	−2,3027
B02	E2F1	E2F transcription factor 1	1,2454
B03	E2F6	E2F transcription factor 6	−1,4777
**B04**	**EGR1**	**Early growth response 1**	**-10,9710**
B05	ELK1	ELK1, member of ETS oncogene family	−1,0743
B06	ESR1	Estrogen receptor 1	−1,1674
B07	ETS1	V-ets erythroblastosis virus E26 oncogene homolog 1 (avian)	−1,5837
B08	ETS2	V-Ets erythroblastosis virus E26 oncogene homolog 2 (avian)	−1,2340
**B09**	**FOS**	**FBJ murine osteosarcoma viral oncogene homolog**	**-5,5533**
B10	FOXA2	Forkhead box A2	2,5198
B11	FOXO1	Forkhead box O1	−1,3044
B12	GATA1	GATA binding protein 1 (globin transcription factor 1)	−1,9498
C01	GATA2	GATA binding protein 2	1,0842
C02	GATA3	GATA binding protein 3	−1,4777
C03	GTF2B	General transcription factor IIB	−1,2340
C04	GTF2F1	General transcription factor IIF, polypeptide 1, 74kDa	−1,2599
C05	HAND1	Heart and neural crest derivatives expressed 1	1,4506
C06	HAND2	Heart and neural crest derivatives expressed 2	2,4566
C07	HDAC1	Histone deacetylase 1	1,0918
C08	HIF1A	Hypoxia inducible factor 1, alpha subunit (basic helix-loop-helix transcription factor)	−1,2426
C09	HNF4A	Hepatocyte nuclear factor 4, alpha	1,0842
C10	HOXA5	Homeobox A5	1,0842
C11	HSF1	Heat shock transcription factor 1	1,0918
C12	ID1	Inhibitor of DNA binding 1, dominant negative helix-loop-helix protein	−2,0326
D01	IRF1	Interferon regulatory factor 1	1,0693
D02	JUN	Jun proto-oncogene	2,5198
D03	JUNB	Jun B proto-oncogene	−1,3883
D04	JUND	Jun D proto-oncogene	−1,1045
D05	MAX	MYC associated factor X	−1,1514
D06	MEF2A	Myocyte enhancer factor 2A	−1,2426
D07	MEF2B	Myocyte enhancer factor 2B	−1,4473
D08	MEF2C	Myocyte enhancer factor 2C	2,9214
D09	MYB	V-myb myeloblastosis viral oncogene homolog (avian)	1,0116
D10	MYC	V-myc myelocytomatosis viral oncogene homolog (avian)	−1,0595
D11	MYF5	Myogenic factor 5	1,0842
D12	MYOD1	Myogenic differentiation 1	1,0842
E01	NFAT5	Nuclear factor of activated T-cells 5, tonicity-responsive	−1,2255
E02	NFATC1	Nuclear factor of activated T-cells, cytoplasmic, calcineurin-dependent 1	−1,4777
E03	NFATC2	Nuclear factor of activated T-cells, cytoplasmic, calcineurin-dependent 2	−1,0595
E04	NFATC3	Nuclear factor of activated T-cells, cytoplasmic, calcineurin-dependent 3	−1,2775
E05	NFATC4	Nuclear factor of activated T-cells, cytoplasmic, calcineurin-dependent 4	−1,0892
E06	NFKB1	Nuclear factor of kappa light polypeptide gene enhancer in B-cells 1	1,2283
E07	NFYB	Nuclear transcription factor Y, beta	−1,0595
E08	NR3C1	Nuclear receptor subfamily 3, group C, member 1 (glucocorticoid receptor)	−1,1674
E09	PAX6	Paired box 6	1,0046
**E10**	**POU2AF1**	**POU class 2 associating factor 1**	**-3,3714**
E11	PPARA	Peroxisome proliferator-activated receptor alpha	−1,0305
E12	PPARG	Peroxisome proliferator-activated receptor gamma	1,2805
F01	RB1	Retinoblastoma 1	−1,2512
F02	REL	V-rel reticuloendotheliosis viral oncogene homolog (avian)	−1,4777
F03	RELA	V-rel reticuloendotheliosis viral oncogene homolog A (avian)	−1,0449
F04	RELB	V-rel reticuloendotheliosis viral oncogene homolog B	1,2030
F05	SMAD1	SMAD family member 1	−1,4880
F06	SMAD4	SMAD family member 4	−1,0234
F07	SMAD5	SMAD family member 5	1,2114
F08	SMAD9	SMAD family member 9	−1,1277
F09	SP1	Sp1 transcription factor	−1,2170
F10	SP3	Sp3 transcription factor	−1,0595
F11	STAT1	Signal transducer and activator of transcription 1, 91kDa	1,0046
F12	STAT2	Signal transducer and activator of transcription 2, 113kDa	−1,2864
**G01**	**STAT3**	**Signal transducer and activator of transcription 3 (acute-phase response factor)**	**-3,1748**
G02	STAT4	Signal transducer and activator of transcription 4	−1,0595
G03	STAT5A	Signal transducer and activator of transcription 5A	−1,2086
G04	STAT5B	Signal transducer and activator of transcription 5B	2,5198
G05	STAT6	Signal transducer and activator of transcription 6, interleukin-4 induced	1,0257
G06	TBP	TATA box binding protein	−1,1755
G07	HNF1A	HNF1 homeobox A	1,0842
G08	TCF7L2	Transcription factor 7-like 2 (T-cell specific, HMG-box)	1,0842
G09	TFAP2A	Transcription factor AP-2 alpha (activating enhancer binding protein 2 alpha)	2,4061
G10	TGIF1	TGFB-induced factor homeobox 1	1,0473
G11	TP53	Tumor protein p53	1,0046
G12	YY1	YY1 transcription factor	−1,2426

For each condition (untreated/treated with atorvastatin) a pool of RNA was constituted, starting with the same amount of RNA (500 ng) from each patient of Cohort 1 (see Methods).

Data are presented as fold of changes in gene expression induced by atorvastatin treatment as compared with the gene expression in untreated CD4^+^T-cells. Increased genes are indicated in red and decreased genes in green. A fold change in gene expression of ≥ 3 was taken as significant.

**Table 3:** Genes investigated by Human Th1-Th2-Th3 RT^2^ Profiler™ PCR Array, and changes in their expression induced by atorvastatin

Position	Symbol	Description	Fold Regulation
A01	IL17A	Interleukin 17A	−1,0822
A02	CCL11	Chemokine (C-C motif) ligand 11	1,0622
A03	CCL5	Chemokine (C-C motif) ligand 5	−1,1696
A04	CCL7	Chemokine (C-C motif) ligand 7	1,0822
**A05**	**CCR2**	**Chemokine (C-C motif) receptor 2**	**-3,4822**
A06	CCR3	Chemokine (C-C motif) receptor 3	2,5974
A07	CCR4	Chemokine (C-C motif) receptor 4	1,0310
A08	CCR5	Chemokine (C-C motif) receptor 5	−1,6656
A09	CD28	CD28 molecule	−1,1859
A10	CD4	CD4 molecule	−2,0648
A11	CD40LG	CD40 ligand	−2,6500
A12	IL23A	Interleukin 23, alpha subunit p19	−1,4804
B01	CD80	CD80 molecule	1,0098
**B02**	**CD86**	**CD86 molecule**	**-7,4436**
B03	CEBPB	CCAAT/enhancer binding protein (C/EBP), beta	−1,2025
B04	CREBBP	CREB binding protein	1,0973
B05	CSF2	Colony stimulating factor 2 (granulocyte-macrophage)	1,0822
B06	CTLA4	Cytotoxic T-lymphocyte-associated protein 4	1,0381
B07	CXCR3	Chemokine (C-X-C motif) receptor 3	−1,1065
B08	FASLG	Fas ligand (TNF superfamily, member 6)	−1,5648
B09	GATA3	GATA binding protein 3	−1,2710
B10	GFI1	Growth factor independent 1 transcription repressor	−1,3250
B11	GLMN	Glomulin, FKBP associated protein	−1,5433
B12	GPR44	G protein-coupled receptor 44	−1,9807
C01	HAVCR2	Hepatitis A virus cellular receptor 2	−1,7484
C02	ICOS	Inducible T-cell co-stimulator	−1,2277
C03	IFNG	Interferon, gamma	−1,7487
C04	IGSF6	Immunoglobulin superfamily, member 6	−2,2439
C05	IL10	Interleukin 10	1,9411
C06	IL12B	Interleukin 12B (natural killer cell stimulatory factor 2, cytotoxic lymphocyte maturation factor 2, p40)	−1,1859
C07	IL12RB2	Interleukin 12 receptor, beta 2	−1,1519
C08	IL13	Interleukin 13	−1,0396
**C09**	**IL13RA1**	**Interleukin 13 receptor, alpha 1**	**-3,6200**
C10	IL15	Interleukin 15	−1,6313
**C11**	**IL18**	**Interleukin 18 (interferon-gamma-inducing factor)**	**-5,8401**
C12	IL18R1	Interleukin 18 receptor 1	−1,1696
D01	IL1R1	Interleukin 1 receptor, type I	−1,1455
D02	IL1R2	Interleukin 1 receptor, type II	1,2693
D03	IL2	Interleukin 2	−2,3554
D04	IL2RA	Interleukin 2 receptor, alpha	−1,4702
D05	IL4	Interleukin 4	1,0822
D06	IL4R	Interleukin 4 receptor	−1,2108
D07	IL5	Interleukin 5 (colony-stimulating factor, eosinophil)	−1,7243
**D08**	**IL6**	**Interleukin 6 (interferon, beta 2)**	**-5,9628**
D09	IL6R	Interleukin 6 receptor	−1,2277
D10	IL7	Interleukin 7	−1,3159
D11	IL9	Interleukin 9	1,0822
D12	INHA	Inhibin, alpha	1,0822
E01	INHBA	Inhibin, beta A	1,4682
E02	IRF1	Interferon regulatory factor 1	1,1761
E03	IRF4	Interferon regulatory factor 4	−1,0324
E04	JAK1	Janus kinase 1	−1,0324
E05	JAK2	Janus kinase 2	−1,0614
E06	LAG3	Lymphocyte-activation gene 3	−1,4103
E07	LAT	Linker for activation of T cells	1,0238
E08	MAF	V-maf musculoaponeurotic fibrosarcoma oncogene homolog (avian)	−1,0468
E09	MAP2K7	Mitogen-activated protein kinase kinase 7	−1,1942
E10	MAPK8	Mitogen-activated protein kinase 8	1,0028
E11	NFATC1	Nuclear factor of activated T-cells, cytoplasmic, calcineurin-dependent 1	−1,3159
E12	NFATC2	Nuclear factor of activated T-cells, cytoplasmic, calcineurin-dependent 2	−1,0468
F01	NFATC2IP	Nuclear factor of activated T-cells, cytoplasmic, calcineurin-dependent 2 interacting protein	−1,2799
F02	PCGF2	Polycomb group ring finger 2	−1,7005
F03	PTPRC	Protein tyrosine phosphatase, receptor type, C	−1,1696
**F04**	**SFTPD**	**Surfactant protein D**	**3,1037**
F05	SOCS1	Suppressor of cytokine signaling 1	1,0098
F06	SOCS2	Suppressor of cytokine signaling 2	−1,5977
F07	SOCS5	Suppressor of cytokine signaling 5	1,0747
**F08**	**SPP1**	**Secreted phosphoprotein 1**	**-5,2634**
F09	STAT1	Signal transducer and activator of transcription 1, 91kDa	1,0673
F10	STAT4	Signal transducer and activator of transcription 4	−1,0973
F11	STAT6	Signal transducer and activator of transcription 6, interleukin-4 induced	1,0028
F12	TBX21	T-box 21	−1,0913
G01	TFCP2	Transcription factor CP2	−1,3813
G02	TGFB3	Transforming growth factor, beta 3	1,1127
**G03**	**TLR4**	**Toll-like receptor 4**	**-3,4822**
G04	TLR6	Toll-like receptor 6	−1,4702
G05	TMED1	Transmembrane emp24 protein transport domain containing 1	−1,4499
G06	TNF	Tumor necrosis factor	1,0973
G07	CD27	CD27 molecule	−1,2025
**G08**	**TNFRSF8**	**Tumor necrosis factor receptor superfamily, member 8**	**-5,4869**
G09	TNFRSF9	Tumor necrosis factor receptor superfamily, member 9	−1,5757
G10	TNFSF4	Tumor necrosis factor (ligand) superfamily, member 4	1,0098
G11	TYK2	Tyrosine kinase 2	−1,1535
G12	YY1	YY1 transcription factor	−1,3909

For each condition (untreated/treated with atorvastatin) a pool of RNA was constituted, starting with the same amount of RNA (500 ng) from each patient of Cohort 1 (see Methods).

Data are presented as fold of changes in gene expression induced by atorvastatin treatment as compared with the gene expression in untreated CD4+T-cells. Increased genes are indicated in red and decreased genes in green. A fold change in gene expression of ≥ 3 was taken as significant.

**Figure 6:**
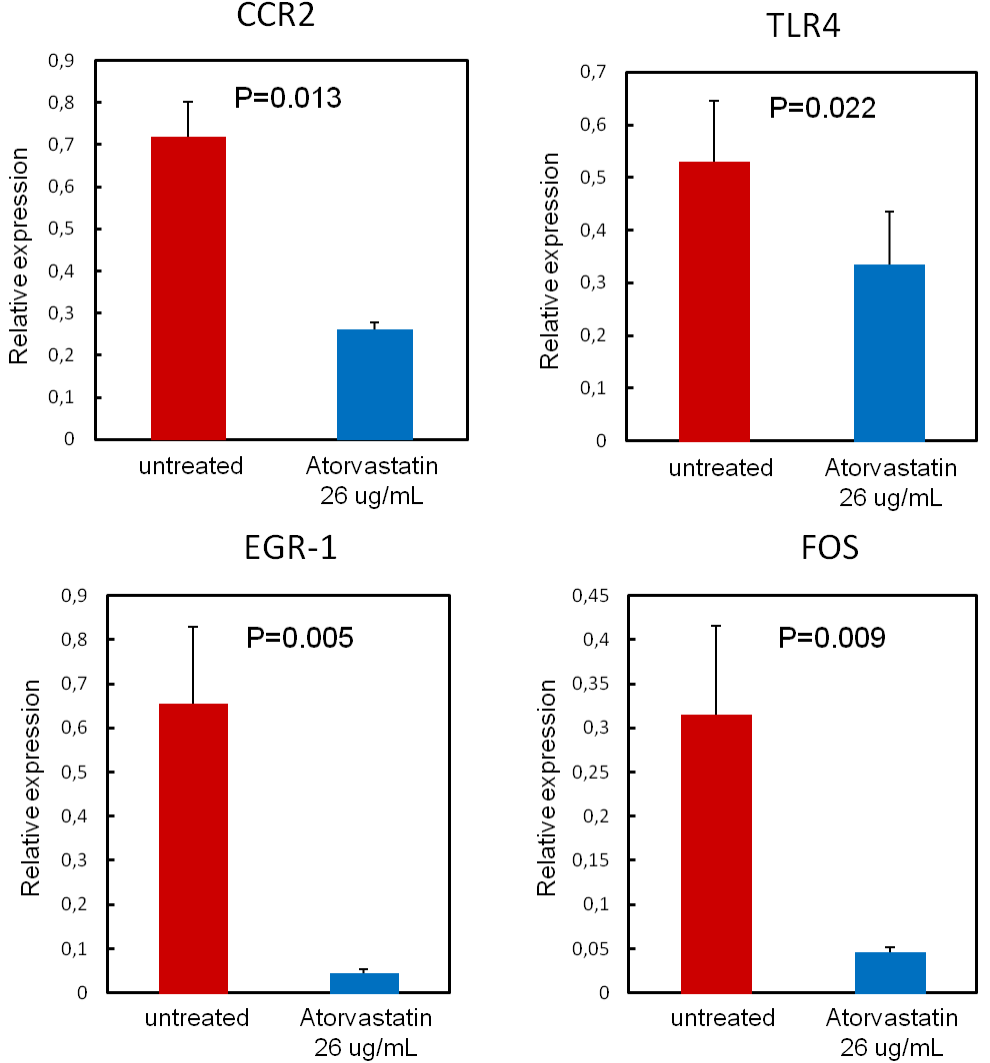
Validation of PCR array results To validate the PCR array results for selected genes, RT-qPCR was performed using single patients RNA (*n* = 20 patients). Data are presented as relative expression compared to human β-2-microglobulin (β-2MG) and glyceraldehyde-3-phosphate dehydrogenase (GAPDH) mRNA levels as endogenous controls, and expressed as mean±SD. Atorvastatin treatment (26 μg/ml for 24hours) significantly decreases the expression of the transcription factors EGR1 (from 0.65±0.18 to0.04±0.01; *P* = 0.005) and FOS (from 0.31±0.10 to 0.05±0.01; *P* = 0.009), Moreover, atorvastatin decreases the expression of the chemokine receptor CCR2 (from 0.72±0.08 to 0.26±0.02; *P* = 0.013) and of the pattern recognition receptor TLR4 (from 0.53±0.12 to0.34±0.10; *P* = 0.022).

To validate the PCR array results for selected genes, RT-qPCR was performed using RNA of each single patient. This set of data confirmed that atorvastatin significantly decreased the expression of CCR2 and of TLR4 as well as the expression of the transcription factors EGR1, FOS (Figure-6).

**Figure 7:**
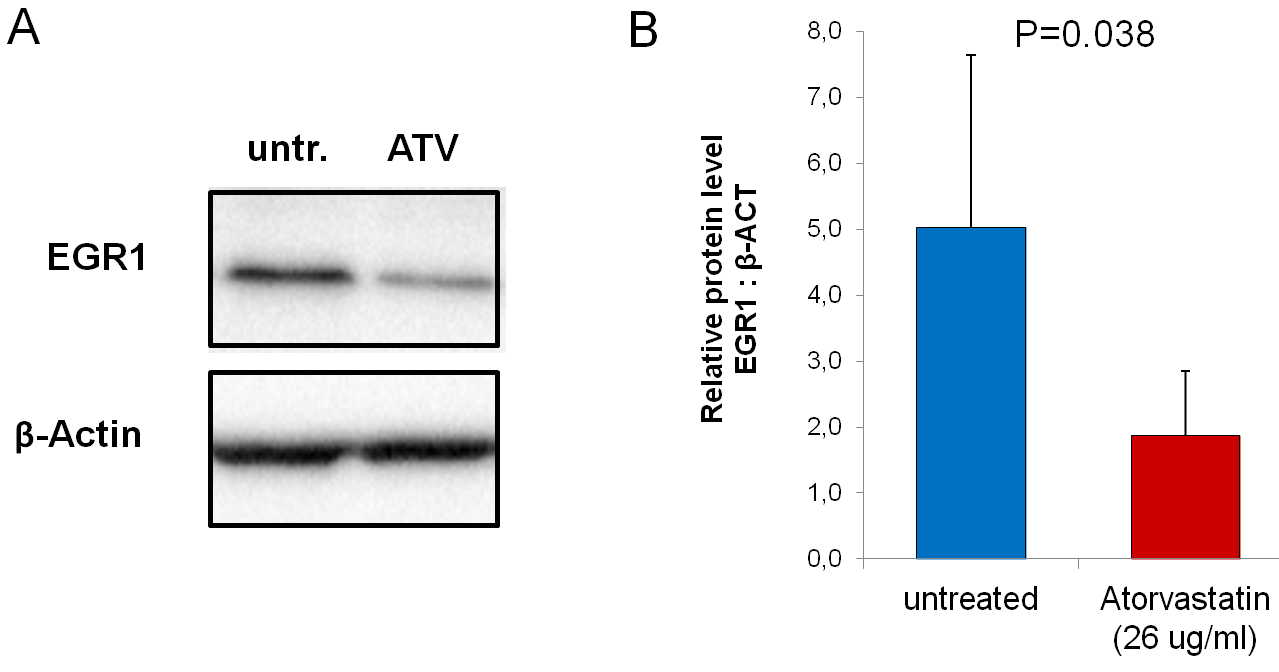
Effect of atorvastatin on EGR-1 protein expression CD4^+^T-cells were cultured for 24 hours with (ATV) and without (untr.) atorvastatin (26μg/ml). Western blot was performed using whole-cell extracts (25 μg per lane) (*n* = 20 patients). **A**. Representative bands for Egr-1 and β-actin loading controls. **B**. After atorvastatin treatment, Egr-1 protein decreased significantly. Data are shown as mean±S.E.M.

We also explored whether atorvastatin treatment might decrease EGR1 protein expression levels, as EGR1 gene showed the highest inhibition at PCR array analysis and also because EGR1 is a transcription factors critically involved in the immune response. Western-blot assay confirmed that incubation with atorvastatin (26 μg/ml) resulted in a significant reduction of EGR1 protein levels (from 5.0±2.6 to1.9±0.97; *P* = 0.038) (Figure-7).

### *In vivo* effects of Atorvastatin

**Figure 8:**
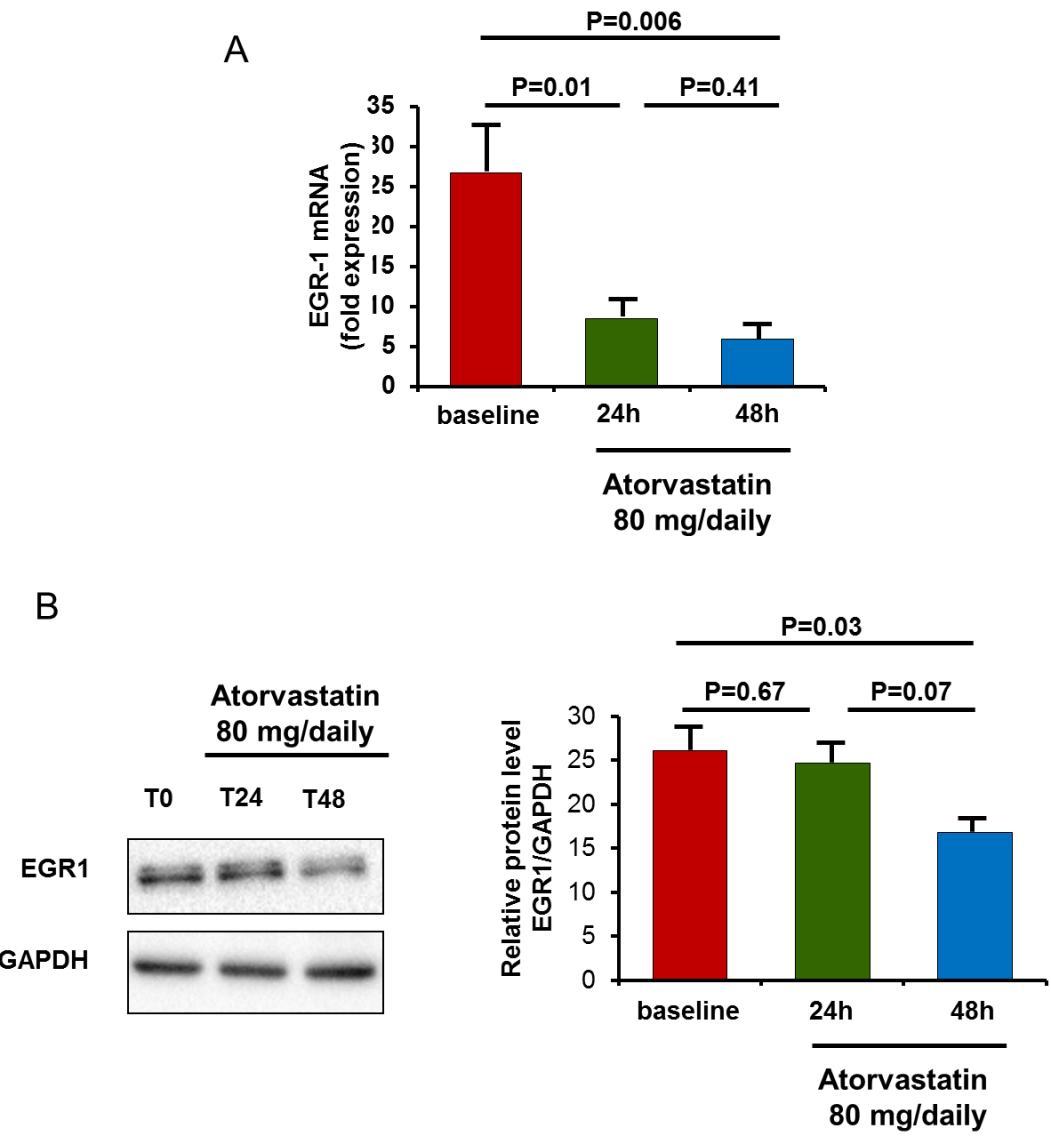
***In-vivo*** effect of atorvastatin on EGR1 gene expression and protein levels. EGR1 gene expression and protein levels were assessed in 10 statin-naïve ACS patients after 24 hours and 48 hours of therapy with atorvastatin (80 mg/daily). **A**. To analyze atorvastatin effects on EGR1 gene expression, RT-qPCR was performed using RNA of each single patient of Cohort 2. Data are presented as relative expression compared to human β-2-microglobulin (β-2MG) and glyceraldehyde-3-phosphate dehydrogenase (GAPDH) mRNA levels as endogenous controls, and expressed as mean±S.E.M. In all patients, EGR1 mRNA levels decreased significantly after 24 hours of atorvastatin therapy. **B**. To analyze atorvastatin effects on EGR1 protein levels, Western blot was performed using whole-cell extracts (25 μg per lane). Representative bands for EGR11 and GAPDH loading controls are shown. After 48 hours of atorvastatin therapy, EGR1 protein decreased significantly. Data are shown as mean±S.E.M.

The *in-vivo* effects of atorvastatin were analyzed in 10 statin-free ACS patients at baseline, and after 24 hours and 48 hours of atorvastatin therapy (80 mg/daily). A single high-dose of atorvastatin has early effects on EGR1 mRNA expression and, with a reasonable delay, on EGR1 protein levels. In all patients, EGR1 gene expression was reduced after 24h of atorvastin therapy, from a mean (± SEM) value of 26.7 ± 5.7 at baseline to 8.5±1.9 at 24 hours (*P* = 0.01 versus baseline) and to 5.9±2.1 at 48hours (*P* = 0.005 versus baseline). Accordingly, EGR1 protein levels were significantly reduced after 48h of atorvastatin treatment, from a mean (± SEM) value of 26.1±2.2 at baseline to 24.7±1.9 at 24 hours (*P* = 0.67) and to 18.8±1.2 at 48 hours (*P* = 0.03 versus baseline) (Figure 8).

**Figure 9:**
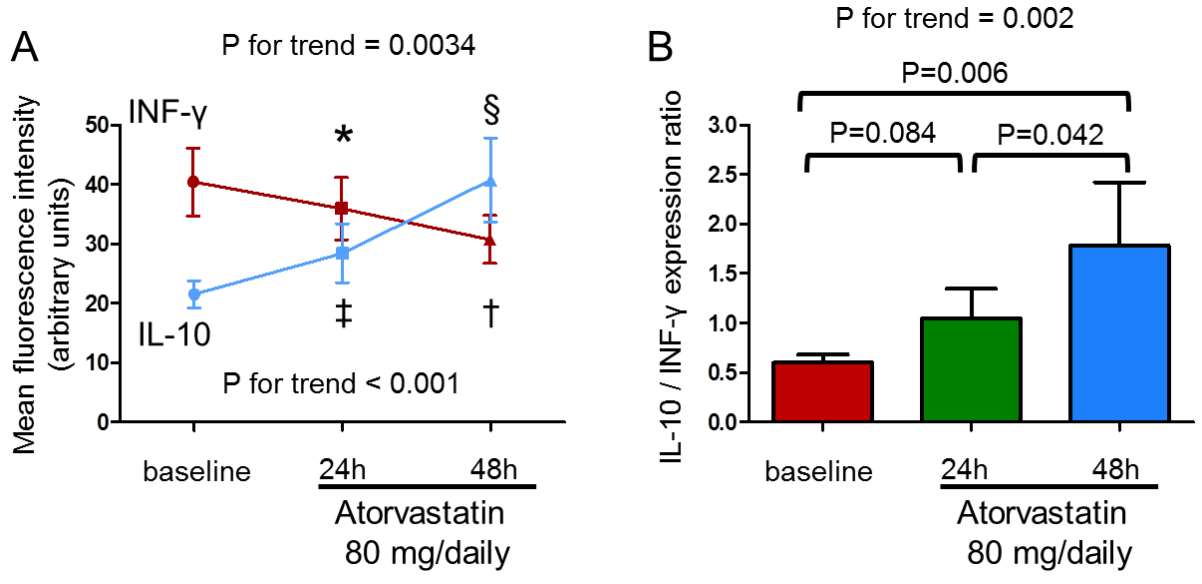
***In-vivo*** effect of atorvastatin on IFN-γ production by CD4**^+^**CD28**^null^**T-cells and IL-10 production by CD4**^+^**CD25^high^T-cells upon stimulation. IFN-γ and IL-10 production were assessed by flow-cytometry in 10 statin-naïve ACS patients after 24 and 48 hours of therapy with atorvastatin (80 mg/daily). **A**. The mean fluorescence intensity of intracellular IFN-γ expression by CD4^+^CD28^null^T-cells significantly decreased (P for trend = 0.0034; *P = 0.12, baseline vs 24 hours; †*P* = 0.010, baseline vs 48 hours; *P* = 0.037, 24 hours vs 48 hours) and the mean fluorescence intensity of intracellular IL-10 expression by CD4^+^CD25^high^T-cells significantly increased after 48hours of atorvastatin therapy (P for trend < 0.001; ‡*P* = 0.16, baseline vs 24 hours; §*P* = 0.004, baseline vs 48 hours; *P* = 0.006, 24 hours vs 48 hours). Data are shown as mean±S.E.M. **B**. Accordingly, the ratio between IL-10/INF-γ expression significantly increased (P for trend = 0.002). Data are shown as mean±S.E.M.

The mean fluorescence intensity of intracellular IFN-γ expression by CD4^+^CD28^null^T-cells significantly decreased after 48hours of atorvastatin therapy (from 40.6±5.7 to 30.9±4.0 mean±S.E.M; P for trend = 0.0034). Moreover, the mean fluorescence intensity of intracellular IL-10 expression by CD4^+^CD25^high^T-cells significantly increased after 48hours of atorvastatin therapy (from 21.5±2.3 to 40.7±7.1; P for trend < 0.001). Accordingly, the ratio between IL-10 and INF-γ expression significantly increased (from 0.59±0.08 to 1.78±0.64; P for trend = 0.002) (Figure 9).

## DISCUSSION

In the present study, we observed that atorvastatin *ex-vivo*, at concentrations corresponding to blood levels achieved with 10-40-80 mg/die, and after short-time (24 hours of incubation), modified the inflammatory activity of T-lymphocytes, although it did not affect their count. Atorvastatin reduced the frequency of CD4^+^CD28^null^T-cells producing IFN-γ, while it increased the production of the anti-inflammatory cytokine IL-10 by CD4^+^CD25^high^T-cells.

We also explored the mechanisms through which atorvastatin might have immune-suppressive effects in ACS, analyzing the expression of relevant transcription factors and genes related to T-cell functional properties. We found that atorvastatin significantly reduced the expression of the chemokine receptor CCR2, involved in trans-endothelial migration of inflammatory cells, and of TLR4. Both CCR2 and TLR4 induce a series of downstream transcriptional factors, including FOS and EGR1, finally leading to the production of a large number of pro-inflammatory cytokines [22, 23, 24]. Furthermore, EGR1 reduces IL-10 expression at the post-transcriptional level [25].

Finally, we explored the *in-vivo* effects of atorvastatin*,* demonstrating that a single high-dose of atorvastatin had also *in-vivo* early effects on EGR1 mRNA expression and on EGR1 protein levels. Moreover, it reduced the frequency of CD4^+^CD28^null^T-cells producing IFN-γ, and increased the production of the anti-inflammatory cytokine IL-10 by CD4^+^CD25^high^T-cells.

### Effects of atorvastatin on CD4^+^T-cell functions

In ACS, CD4^+^CD28^null^T-cells are increased in peripheral blood [26] and infiltrate unstable coronary plaques where they undergo clonal expansion [27], probably triggered by specific antigens [28].They produce pro-inflammatory cytokines, in particular IFN-γ [26], and express high levels of TRAIL, a member of the TNF family implicated in apoptosis of vascular smooth muscle cells [29]. By directly stimulating apoptosis of vascular smooth muscle cells or by activating macrophages to kill these ones trough IFN-γ production, CD4^+^CD28^null^T-cells could weaken the fibrous cap and destabilize angiogenic vessels, precipitating atherosclerotic plaque rupture [30]. Moreover, these T-cells spontaneously express the subunit β1 of the IL-12 receptor even in the absence of antigenic stimulation, and respond to direct IL-12 stimulation with an increased expression of chemokine receptors that promote the tissue homing of effector T-cells [31]. In contrast, the anti-inflammatory cytokine IL-10 contributes to the atheroprotective effects of regulatory T-cells [32], and the expression of regulatory T-cells co-localizes with IL-10 within the atherosclerotic plaques [33]. ACS patients have a skewed helper T-cell differentiation oriented towards the expansion of aggressive effector T-cells and the reduction of Treg number and function [34]. These helper T-cell abnormalities characterize a sizeable proportion of ACS patients [10]. In this subset of ACS patients, the immune response might contribute to plaque destabilization through multiple damaging pathways [35].

Short-term treatment of ACS patients with statins is associated with a reduction of inflammatory markers [36], and with a rapid reduction in the intracellular production of TNF-α and IFN-γ by T-cells in-vitro [37]. Rosuvastatin, fluvastatin, and pitavastatin *in-vitro* treatment inhibits CD4^+^T-cell-induced endothelial cell apoptosis by suppressing T-cell activation and TRAIL expression upon activation [38]. In two previous observational reports we found that the use of statins was associated with reduced levels of CD4^+^CD28^null^T cells [17, 2].

Furthermore, short-term treatment of ACS patients with statins is associated with a significant increase of regulatory T-cell inhibitory properties and a significant reduction of serum IFN-γ and increase of IL-10 [39, 40]. Statins may enhance regulatory T-cell responses by promoting their chemokine-dependent recruitment into inflammatory sites [21] and/or their differentiation in the periphery [19].

### Effects of atorvastatin on the expression of inflammatory genes and transcription factors involved in the immune response

In our study atorvastatin reduced TLR4 gene expression, a pattern-recognition receptor stimulated by several pathogen associated molecular patterns (PAMPs) and damage associated molecular patterns (DAMPs). TLR4 has been found in atherosclerotic lesions and at the site of plaque rupture in patients with MI [41]; its expression is increased in thrombi [42] and in circulating monocytes [43] from patients with ACS. TLR4 stimulation induces intracellular pathways converging on nuclear factor (NF)-kB and mitogen activated protein kinases (MAPK), with subsequent release of pro-inflammatory cytokines and expression of co-stimulatory molecules [23].

**Figure 10:**
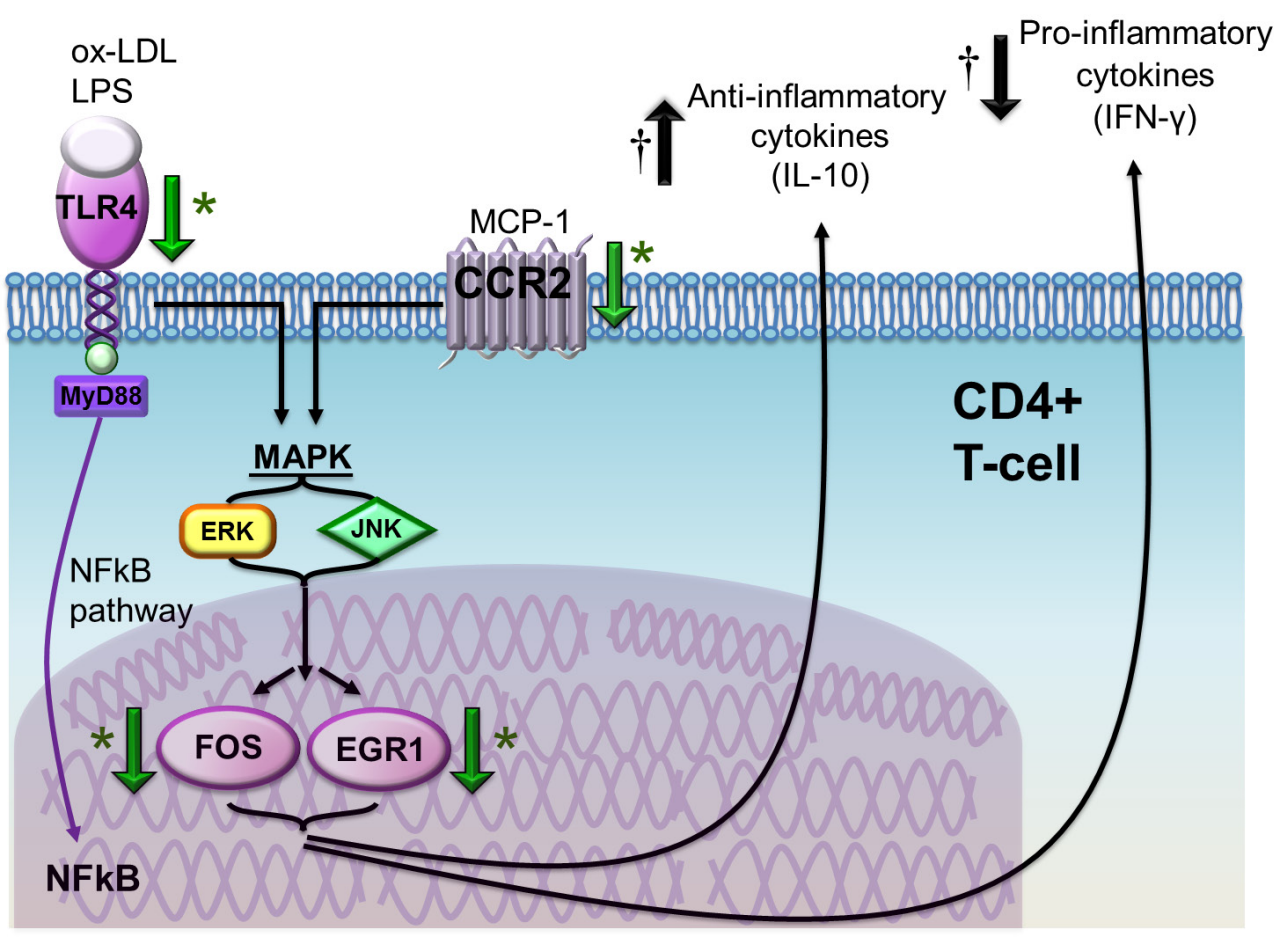
Schematic representation of the effects of atorvastatin on CD4^+^ ^T-cells in patients with ACS.^ Atorvastatin decreases toll like receptor (TLR)-4 gene expression, a pattern-recognition receptor stimulated by several pathogen-associated molecular patterns (PAMPs) and damage associated molecular patterns (DAMPs), including bacterial lipolysaccaride (LPS) and ox-LDL, that has been implicated in the initiation and progression of atherosclerosis. TLR-4 stimulation induces intracellular pathways converging on nuclear factor (NF)-kB and mitogen-activated protein kinases (MAPK), with subsequent release of pro-inflammatory cytokines and expression of co-stimulatory molecules. Atorvastatin also reduces the expression of the chemokine (C-C motif) receptor 2 (CCR2), which is the receptor for monocyte chemoattractant protein (MCP)-1 and is involved in CD4^+^T-cell transendothelial migration and recruitment at the site of tissue damage and inflammation. CCR2 intracellular pathway also converges on MAPK pathway, resulting in the activation of ERK and JNK and, eventually, of nuclear transcription factors FBJ murine osteosarcoma viral oncogene homolog (FOS) and early growth response 1 (EGR1), implicated in the immune response. Indeed, we observed an important decrease of EGR1 gene and protein expression by a single high-dose of atorvastatin both *ex-vivo* and *in-vivo*, suggesting a specific direct effects of atorvastatin on EGR1. The final net effect is a reduction of pro-inflammatory cytokine secretion and of chemokine and chemokine receptor synthesis, and an increase of anti-inflammatory pathways. *Green arrows indicate the effects of atorvastatin according to molecular assays (reduced expression of the TLR4, CCR2, FOS and EGR1 genes and of EGR1 protein); † black arrows indicate the opposite effects of atorvastatin on IFN-γ (reduced intracellular expression by CD4+CD28nullT-cells) and IL-10 (increased intracellular expression by CD4+CD25highT-cells) as assessed by flow-cytometry.

Atorvastatin also reduced the expression of the chemokine receptor CCR2, that is involved in CD4^+^T-cell transendothelial migration and recruitment at the site of tissue damage and inflammation [23]. CCR2 intracellular pathway also converges on MAPK and, downstream, on the nuclear transcription factors FOS and EGR1, both implicated in the immune response [22]. Indeed, we observed a decrease of the expression of these two transcription factors by atorvastatin. In particular, EGR1 gene showed the highest inhibition (Figure 10).

EGR1 is the prototype of a family of zinc-finger transcription factors, “immediate-early response proteins”, that is rapidly and transiently induced by a broad spectrum of extracellular signals, including growth factors, cytokines, injurious stimuli and many physiologic stimuli [44]. EGR1 is involved in cell growth, cell differentiation and cell survival [45]. EGR1 is expressed in T-cells and promotes T-cell activation and development by transcriptional induction of key cytokines, such as IL-2 and TNF-α, and costimulatory molecules, such as CD40 ligand, after T-cell receptor (TCR) stimulation [46, 22]. EGR1 also induces the transcriptional activation of T-bet, the master gene regulator of Th1 [47], a T-cell subsets involved in atherosclerosis progression and plaque destabilization [48].

Conversely, EGR1 reduces the expression of IL-10 at post-transcriptional level, by inducing the transcription of a microRNA (hsa-miR-106a) that degrades IL-10 mRNA [25]. EGR1 might also promote atherogenesis through the activation of inflammatory genes [49, 50]. Thus, we could speculate that, by reducing the expression of the transcription factor EGR1 in CD4+ T-cells, atorvastatin treatment might have the final net effect of reducing pro-inflammatory cytokine secretion, chemokine and chemokine receptor synthesis. This hypothesis is strongly supported by the *in-vivo* observation that a single high-dose of atorvastatin has early effects on EGR1 mRNA expression and, with a reasonable delay, on EGR1 protein levels. Moreover, it modifies the inflammatory profile of T-lymphocytes by decreasing IFN-γ production by aggressive effector T-cells, and increasing the production of the anti-inflammatory cytokine IL-10 by T-cells with a regulatory phenotype (Figure 10).

Although some of these beneficial effects have already been attributed to different statins in humans [51, 52] as well as in animal models [53] and *in-vitro* [54], our study investigates a comprehensive expression profile in ACS.

In the setting of ACS, it has been proposed that the early outcome improvement observed with intensive statin treatment, compared to a moderate treatment schedule, might be related to anti-inflammatory properties rather than to lipid-lowering effects. According to this, the improvement of short-term outcome associated to intensive statin treatment appeared to correlate with hs-CRP level reduction rather than with LDL-cholesterol level lowering [55]. However, hs-CRP is more likely a risk marker rather than a causal factor of atherosclerosis [56, 57]. The current data show that atorvastatin acts on the immune-response, offering a causal explanation on why statins ameliorate prognosis in ACS.

## CONCLUSIONS

In ACS, ex-vivo atorvastatin treatment decreases the expression of transcription factors in T-cells, in particular the “immediate-early response protein” EGR1, resulting in inhibition of pro-inflammatory effector T-cells and activation of anti-inflammatory T-cells. EGR1 reduction by atorvastatin has also been confirmed *in-vivo*, after a single high-dose treatment. Therefore, in the setting of ACS, the early outcome improvement of intensive statin treatment might, at least partially, be related to direct inhibition of the master regulator EGR1 and to consequent immune-suppressive effects.

Some of the pathways inhibited by atorvastatin in the present study have recently been proposed as new therapeutic targets for the prevention of cardiovascular diseases. This is the case for the MCP-1/CCR2 pathway [58], the TLR4 pathway [59], and for a newly identified microRNA that functions as a negative regulator on inflammatory cytokines TNF-α and IL-6 via targeting EGR1 in vivo [60].

## MATERIALS AND METHODS

### Study population

We prospectively evaluated 334 consecutive patients admitted to our CCU from June 2011 to June 2013 with a diagnosis of NSTE-ACS. We enrolled 40 patients (Cohort 1) who had never received statin treatment and with circulating CD4^+^CD28^null^T-cell frequency >4%, as our group has previously shown that CD4^+^CD28^null^T-cell frequency >4% predicts recurrence of acute coronary events [2]. Ten additional patients (Cohort 2) were enrolled from November 2013 to May 2014 using the same inclusion criteria, to analyze the *in-vivo* effects of atorvastatin on EGR1 gene expression and protein levels.

Exclusion criteria were: 1) age >80 years; 2) evidence of inflammatory or infectious diseases, malignancies, immunologic or hematological disorders; 3) diabetes mellitus; 4) ejection fraction < 40%; 5) treatment with anti-inflammatory drugs other than low-dose aspirin.

The study was approved by the local ethics committee and appropriate consent was obtained from study patients.

### Screening for CD4^+^CD28^null^T-cell frequency

Immediately after CCU admission, 1 ml of whole blood anti-coagulated with EDTA was used to assess CD4^+^CD28^null^T-cell frequency by flow cytometry, using anti-CD4 fluorescin-isothiocyanate (FITC) conjugated and anti-CD28 phycoerythrin-Cy5 (PE-Cy5) conjugated monoclonal antibodies (all Beckman Coulter, Brea, CA). After the initial screening, venous peripheral blood samples were obtained from patients with circulating CD4^+^CD28^null^T-cell frequency >4%.

### Isolation of CD4^+^T-cells and atorvastatin treatment

Peripheral blood mononuclear cells (PBMCs) were obtained from whole blood samples by standard gradient centrifugation over Ficoll-Hypaque (GE Healthcare Bio-Sciences, Piscataway, NJ). CD4^+^T-cells were isolated by magnetic micro-beads (CD4+T-cell isolation kit MACS, Miltenyi Biotec, Auburn, CA) according to the manufacturer's instructions. Pure atorvastatin, kindly provided by Pfizer (New York, NY), was dissolved in 2% dimethilsulfoxide (DMSO) solution at final concentration of 50mM. CD4^+^T-cells were incubated for 24 hours, under sterile conditions at 37°C in an atmosphere containing 5% CO2, at a density of 1 × 10^6^/ml in RPMI 1640 medium (Sigma, St. Louis, MO) supplemented with 10% fetal bovin serum (Invitrogen, Carlsbad, CA) with and without increasing doses of atorvastatin: 3-10-26 μg/ml. These doses were chosen on the basis of the observation that comparable plasmatic concentrations correspond to in vivo doses of 10-40-80 mg of atorvastatin [61].

### T-cell analysis by flow cytometry

In Cohort 1 patients, phenotypic and functional characteristics of CD4^+^T-cells treated/untreated with atorvastatin were assessed by flow-cytometry using fluorochrome-conjugated monoclonal anti-human antibody (mAb), including isotype controls. Flow-cytometry quantization was performed on 40.000 live cells. Data acquisition was performed using a FC500 Flow Cytometry System (Beckman Coulter, Brea, CA) and data analysis using the Kaluza^®^ analysis software packages (Beckman Coulter, Brea, CA).

We assessed total CD4+, CD4+CD28null, CD4+CD25high, CD4+CD25highFoxp3+T-cells. We also determined IFN-γ production by CD4+CD28nullT-cells and IL-10 production by CD4+CD25highT-cells upon stimulation.

CD4^+^CD28^null^ T-cell frequency was determined using anti-CD4-fluorescinisothiocyanate (FITC)-conjugated mAb and anti-CD28-phycoerythrin-Cy5(PE-Cy5)-conjugated mAb (both Beckman Coulter, Brea, CA). The percentage of CD4^+^CD28^null^T-cells was expressed as percentage of the entire population of CD4^+^T-cells.

CD4^+^CD25^high^T-cell frequency was obtained using monoclonal anti-CD4-FITC-labeled and monoclonal anti-human CD25-APC-labeled, on the basis of high CD25-APC fluorescence in comparison with intermediate CD25-APC fluorescence in CD4^+^CD25^+^T-cells. After cell surface staining, cell fixation and permeabilization, cells were stained with the intracellular PE-conjugated anti-Foxp3 mAB (eBioscience, San Diego, CA). The percentage of CD4^+^CD25^high^T-cells and of CD4^+^CD25^high^T-cells expressing Foxp3^+^T-cells was then calculated as percentage of CD4^+^CD25^+^T-cell population.

In order to evaluate T-cell function, we determined IFN-γ production by CD4^+^CD28^null^T-cells and IL-10 production by CD4^+^CD25^high^T-cells upon stimulation. In the assessment of IL-10 production, regulatory T-cells were identified as CD4^+^CD25^high^ T-cells, in order to avoid a stressful cell-handling as required to perform permeabilization for intra-cytoplamasmatic and intra-nuclear protein assessment, potentially resulting in a less accurate measurement of IL-10. A significant correlation was observed between CD4^+^CD25^high^T-cells and CD4^+^CD25^high^ Foxp3^+^T-cells (R = 0.67; *P* < 0.001). Briefly, cytokines production by CD4^+^T-cell subsets was assessed after 4-hours in vitro activation with 100 ng/ml phorbol-2-myristate-13-acetate (PMA) (Sigma, St. Louis, MO) and 1μg/ml ionomycin (Sigma, St. Louis, MO). Cells were incubated in polypropylene tubes at 37°C for a total of 4 hrs; during the last 2 hrs, 10 μg/mL Brefeldin A (Sigma, St. Louis, MO) was added to block extracellular secretion of cytokines. After cell surface staining, cell fixation was done with IC FIX Buffer (eBioscience, San Diego, CA) for 15 min at 4°C. Cell membranes were reversibly permeabilized with Permeabilization Buffer (eBioscience, San Diego, CA) and intracellular cytokines were labeled with mouse anti-human IFN-γ PE-conjugated (eBioscience, San Diego, CA) and mouse anti-human IL-10 Peridinin-Chlorophyll-Protein-Complex (PerCP)-conjugated (R&D Systems, Minneapolis, MN). In addition, IFN-γ and IL-10 intracellular expression was presented as MFI.

### Cytokine measurements

In the same patients of Cohort 1, citrate whole-blood samples were collected from an antecubital vein at the time of patient enrollment. Immediately after sampling, aliquots of 1 mL of whole blood were incubated for 24 hours under sterile conditions at 37°C in an atmosphere containing 5% CO2 without and with increasing doses of atorvastatin: 3, 10, 26 μg/ml. Afterwards, plasma samples were obtained, stored at −80°C and analysed in a single bath at the end of the study. Plasma levels of IL-10 and IFN-γ were measured with high-sensitivity ELISA kits (human-IL-10, Aushon Biosystems, Billerica, MA; human-IFN-γ, Bender MedSystem, Vienna, Austria), according to the manufacturer's instructions. The linear range of detection was 0.78 to 200 pg/ml for IL-10 and 1.6 to 100 pg/ml for IFN-γ. All samples were measured in duplicate, and the intra- and inter-assay variability was < 10%.

### RNA preparation and quantitative PCR array analysis

Patients in Cohort 1 underwent quantitative PCR array. CD4^+^T-cells were incubated for 24 hours without and with 26 μg/ml of atorvastatin (see above). Total RNA was isolated by using RNeasy minikit (Qiagen, Valencia, CA) from 3×10^6^ CD4^+^T-cells per patient. RNA was quantified using a Picodrop spectrophotometer measuring the absorbance at 260/280 nm, and RNA integrity was confirmed with the Agilent Bioanalyzer (Agilent Technologies, Santa Clara CA). For each condition (untreated/treated with atorvastatin) a pool of RNA was constituted, starting with the same amount of RNA (500 ng) from each patient.

Array targets were prepared from 435 ng of total RNA from each pool (untreated/treated with atorvastatin) using SABiosciences Kit for genomic DNA removal and reverse transcription, and two focused panels of genes were analyze by quantitative PCR array (RT^2^ Profiler™ PCR Array, SABiosciences, Frederick, MD, USA). We performed the Human Transcription Factors RT^2^ Profiler™ PCR Array (Catalog #PAHS-075) and the Human Th1-Th2-Th3 RT^2^ Profiler™ PCR Array (Catalog #PAHS-034) both profiling the expression of 84 genes, according to the manufacturer's instruction. Thermal cycling parameters were 95°C for 10 min, followed by 40 cycles of amplifications at 95°C for 15 s, 55°C for 30 s, 72°C for 30 s, and 72°C for 5 min as the final elongation step performed on IQ5 Icycler (BioRad). Relative levels of mRNA expression were normalized in all samples with the expression levels of housekeeping genes, and data analysis was done using the Web portal (http://www.sabiosciences.com/pcr/arrayanalysis.php). The relative expression of each gene was compared with the expression in the control group and calculated using the ΔΔCT method. Each reported value represented the mean decrease (or increase) of mRNA expression relative to the control levels. A P-value of ≤0.05 and a fold change in gene expression of >3 were taken as significant.

### Quantitative real-time RT-PCR

Confirmation and validation of candidate genes was performed by real time quantitative polymerase chain reaction (RT-qPCR) after reverse transcription of RNA obtained from each single patient of Cohort 1 using the iScript™ cDNA Synthesis Kit (BioRad Laboratories, Hercules, CA). For each patient 250 ng of RNA from CD4^+^T-cells untreated/treated with atorvastatin was used to synthesize cDNA in a final volume of 20 μl. 1 μl of cDNA was used as template for RT-qPCR in a 15 μl reaction mixture, including SsoAdvanced™ SYBR^®^ Green supermix (BioRad) and 400 nm of each primer. Oligonucleotide primers for RT-qPCR were designed with Beacon Design (Table 4). RT-qPCR was performed on triplicates samples using the IQ5 Icycler (BioRad). After initial denaturation step of 30 sec at 95°C, a two-steps cycling procedure (denaturation at 95°C for 5 sec, annealing and extension at 64°C for 30sec) was performed for 40 cycles and followed by melting curve at 95°C for 6 sec. Data are normalized to human β-2-microglobulin (β-2MG) and glyceraldehyde-3-phosphate dehydrogenase (GAPDH) mRNA levels as an endogenous control and are expressed relative to control sample using the formula 2^-ΔΔCT^, where C_T_ is the threshold cycle number.

**Table 4:** Oligonucleotide primers used for real time quantitative polymerase chain reaction

**GeneBank accession number**	**Gene name**	**Primers 5’-3’**
NM_001964	Early growth response (EGR1)	For GAGCAGCCCTACGAGCAC Rev GTCTCCACCAGCACCTTCTC
NM_005252	V-fos FBJ murine osteosarcoma viral oncogene homolog (FOS)	For GACCTTATCTGTGCGTGAA Rev CACTGGGAACAATACACACT
NM_001123396	Chemokine (C-C motif) receptor 2 (CCR2)	For GCATTCAGCCAGGAGATG Rev ATCATCGGACTCCACCAA
NM_138554	Toll-like receptor 4 (TLR4)	For GCCCTGCGTGGAGGTGGTT Rev GGGGAGGTTGTCGGGGATTTTGT
NM_004048	β-2-Microglobulin (B2M)	For AGGACTGGTCTTTCTATCTCTTGT Rev ACCTCCATGATGCTGCTTACA
NM_002046	Glyceraldehyde-3-phosphate dehydrogenase (GAPDH)	For AGTCAGCCGCATCTTCTT Rev GCCCAATACGACCAAATCC

Confirmation and validation of candidate genes among those reduced or increased by atorvastatin was performed by real time quantitative polymerase chain reaction (RT-qPCR) after reverse transcription of single-patient RNA.

Also, the *in-vivo* effect of atorvastatin on EGR1 gene expression was assessed by RT-qPCR.

### Western-blot assays

We also explored whether atorvastatin treatment might influence protein expression levels of EGR1, both *ex-vivo* and *in-vivo*. Western-blot analysis was carried out in CD4^+^T-cells (1×10^6^) cultured for 24 hours with and without 26 μg/ml atorvastatin. Western blot was performed using whole-cell extracts (25 μg per lane). Protein were resolved on 7% SDS-PAGE gels and transferred to nitrocellulose membranes. Membranes were blocked with 5% non-fat milk in phosphate buffered saline and 1% Tween-20 (TBS) and labeled with primary antibodies (all from Santa Cruz Biotechnology, Dallas, Tx): mouse monoclonal IgG_1_ anti-human β-actin (sc-130301) and rabbit polyclonal IgG anti-human EGR1 p82 (588, sc-110). To saving samples, materials, and time we stripped and re-probed a single membrane for multiple targets instead of running and blotting multiple gels. Membranes were stripped with a buffer consisting of β-mercaptoethanol, SDS, and Tris-HCl and re-probed with the specific antibodies. Then, specific bands were detected by a chemiluminescent system (ChemiDoc MP System, Biorad) using corresponding secondary antibodies conjugated with horseradish peroxidase.

Also, the *in-vivo* effect of atorvastatin on EGR1 protein expression was assessed by western-blot.

### *In-vivo* effects of atorvastatin

*In-vivo* effects of atorvastatin on EGR1 gene expression and protein levels were analyzed in Cohort 2 patients treated with atorvastatin 80 mg/daily, at 24 hours and 48 hours of treatment, by RT-qPCR and by western-blotting, respectively (see above).

We also determined the *in-vivo* effects of atorvastatin on IFN-γ production by CD4+CD28nullT-cells and IL-10 production by CD4+CD25highT-cells upon stimulation by flow cytometry (see above).

### Statistical analysis

No power calculation could be performed because of lack of previous studies in this setting. Thus, the enrollment of 40 patients in Cohort 1 and of 10 patients in Cohort 2 was arbitrary.

The percentage of T-cell subsets along with cytokine production by these T-cells, were not normally distributed; they were expressed as median and range and analyzed using the Friedman's two-way analysis of variance by ranks for multiple pairwise comparisons with Dunnet's correction. The remaining continuous variables, including gene and protein expression were normally distributed; they were expressed as mean±SD and were compared using 1-way ANOVA for repeated measures with the Bonferroni correction for multiple pairwise comparisons or using the paired t-test, as appropriate. Proportions were compared using the Chi square test.

Univariate logistic regression analysis was applied to individuate the variables associated with the effects of atorvastatin (a reduction of CD4^+^CD28^null^T-cells producing IFNγ and/or an increase of CD4^+^CD25^high^T-cells producing IL-10 higher than 50% after incubation with atorvastatin 26 μg/ml). The following clinical and laboratory variables were tested: age, sex, classical risk factors, previous history of acute coronary events, left ventricular ejection fraction, multi-vessel disease, troponin T levels, lipid profile (total-cholesterol, LDL-cholesterol, and HDL-cholesterol). At univariate logistic regression analysis none of the variables considered, including lipid profile, demonstrated any association with effects of atorvastatin. Therefore, the multivariate analysis was not performed.

A two-tailed *P* value < 0.05 was considered statistically significant. Statistical analysis was performed with SPSS 18.0 software (SPSS Inc., Chicago, Illinois).

## Abbreviations

INF-γ: Interferon-γ
ACS: Acute Coronary Syndromes
IL-10: Interleukin-10
NSTE: Non-ST Elevation
EGR1: Early Growth Response 1
TLR4: Toll-like Receptor-4
CCR2: Chemokine (C-C motif) Receptor-2
PAMPs: Pathogen Associated Molecular Patterns
DAMPs: Damage Associated Molecular Patterns
TCR: T-cell Receptor
NF-kB: Nuclear Factor-kB
